# Retrospective Evaluation of the Efficacy of Prophylactic Antibiotics in Preventing Surgical Site Infections in Elliptical Skin Biopsy

**DOI:** 10.7759/cureus.83448

**Published:** 2025-05-04

**Authors:** Airin Sato, Yu Matsui, Teruhiko Makino, Tadamichi Shimizu

**Affiliations:** 1 Department of Dermatology, University of Toyama, Toyama, JPN

**Keywords:** elliptical skin biopsy, excisional biopsy, fusiform biopsy, postoperative prophylactic antibiotic, retrospective study, surgical site infections (ssi)

## Abstract

Introduction: Elliptical skin biopsy is a commonly performed dermatologic procedure that provides sufficient tissue for histopathologic diagnosis and additional testing. However, concerns remain regarding the risk of surgical site infection (SSI), leading to the widespread use of prophylactic antibiotics despite the lack of clear supporting evidence. This study aimed to evaluate whether prophylactic antibiotic administration reduces SSI risk following elliptical biopsy.

Methodology: A retrospective observational study was conducted at the University of Toyama, including 202 patients who underwent elliptical biopsy between October 2022 and July 2024. Among them, 146 (72.3%) received prophylactic antibiotics, while 56 (27.7%) did not. The primary outcome was the incidence of SSI within 30 days post procedure. Multivariable logistic regression and inverse probability of treatment weighting (IPTW) were applied to adjust for potential confounders.

Results: SSI occurred at similar rates in both groups (prophylactic vs. non-prophylactic: 11.0% vs. 10.7%, p = 1.0), suggesting that antibiotic prophylaxis may not be effective in reducing infection risk. Neither multivariable logistic regression nor IPTW analysis demonstrated a significant association between prophylactic antibiotic use and SSI prevention. Additionally, clinicians with more years of experience were less likely to prescribe prophylactic antibiotics, indicating greater confidence in withholding antibiotics when not clinically necessary. In contrast, higher serum albumin levels were associated with greater antibiotic use. Although this association reached statistical significance, the actual difference in albumin levels (4.1 vs. 3.8 g/dL) is modest and likely lacks clinical relevance. These findings suggest that antibiotic prescribing may have been influenced more by physician discretion, particularly among less experienced clinicians, as well as by institutional practices, routine habits, or excessive concern about SSI risk, rather than by objective indicators of clinical need.

Conclusion: These findings suggest that routine prophylactic antibiotic use after elliptical biopsy may not be necessary in dermatologic practice. Given increasing concerns about antimicrobial resistance and antibiotic shortages, optimizing antibiotic use is a critical consideration. Emphasizing alternative strategies, such as meticulous wound irrigation, postoperative care, and patient education, may help minimize SSI risk while ensuring safe wound healing. Further prospective studies with larger cohorts and standardized treatment protocols are warranted to confirm these findings.

## Introduction

Dermatology covers a wide range of conditions, including inflammatory diseases, traumatic injuries, and malignant tumors. Among the various procedures performed, skin biopsy, particularly elliptical (or excisional) biopsy, is a fundamental diagnostic tool, commonly used to evaluate deep dermal or subcutaneous lesions, nodular lesions, and suspected malignancies [1-4]. This technique yields sufficient tissue for histopathologic evaluation and ancillary tests such as direct immunofluorescence (DIF).

While skin biopsies are generally considered low-risk, one notable complication is surgical site infection (SSI). The National Healthcare Safety Network (NHSN) defines SSI and highlights contributing factors such as wound characteristics, host comorbidities, anatomical location, and procedural complexity [5,6]. Although the association between wound size and SSI remains debated, larger wounds (e.g., >2 cm) may carry increased risk [7]. Compared to punch biopsy, elliptical biopsy often results in deeper, longer wounds, potentially increasing the likelihood of SSI and its consequences, including antibiotic use, delayed healing, and increased healthcare costs [7,8].

To reduce this risk, some dermatologists routinely prescribe prophylactic antibiotics. For instance, 43% of dermatologists in the United Kingdom report doing so [9]. However, major guidelines such as those by the American College of Surgeons and the Infectious Diseases Society of America generally discourage routine antibiotic use in clean dermatologic procedures [10]. In Japan, relevant dermatological or surgical guidelines also lack specific recommendations for prophylactic antibiotic use in skin biopsies [11], leaving the decision to individual clinicians.

Although antibiotic prophylaxis is usually considered unnecessary for punch biopsies [12], its utility in elliptical biopsies, procedures more invasive than punch but less extensive than major surgeries, remains unclear. Given the potential for layered closures and larger wounds, elliptical biopsy may pose a higher infection risk. To address this gap, we conducted a retrospective study to evaluate whether prophylactic antibiotic use affects the incidence of SSI following elliptical biopsy in a dermatologic setting.

## Materials and methods

This was a retrospective observational study that assessed the efficacy of prophylactic antibiotic administration in reducing the incidence of SSI following elliptical skin biopsy procedures. While these procedures were primarily performed for diagnostic purposes, a subset also carried therapeutic intent, particularly in cases of suspected tumors not requiring wide excision. Many of the patients were seen as first-time visitors referred from other institutions, often following an initial punch biopsy. The study was approved by the Toyama University Hospital Ethics Board, which waived the requirement for patient consent (study number: R2024812), and was conducted in accordance with the Declaration of Helsinki.

Study population

Patients who underwent elliptical skin biopsies between October 2022 and July 2024 at the Department of Dermatology, University of Toyama, were included. Patients who underwent biopsies for multiple lesions or had a pre-existing local infection at the biopsy site (including ulcerated or obviously infected lesions) were excluded to ensure consistent evaluation of postoperative outcomes.

A total of 202 patients were included, of which 146 received perioperative antibiotic prophylaxis, while 56 did not.

Data collection and variables

The primary outcome measure was the presence or absence of SSI within 30 days post procedure. SSI occurrence was evaluated during suture removal (7-14 days post biopsy), at the follow-up appointments for pathology results (approximately four weeks post procedure), or during any subsequent follow-up within the first month. SSI was defined according to the criteria outlined by the NHSN in 2024 [5]. Specifically, a superficial incisional SSI was diagnosed when the infection was confined to the skin or subcutaneous tissue and met at least one of the following clinical criteria: purulent discharge from the incision, isolation of a pathogen from an aseptically collected specimen, deliberate incision reopening due to localized symptoms (pain, swelling, erythema, or warmth), or a physician’s diagnosis of SSI. Cases involving cellulitis without drainage, stitch abscesses, and localized stab wounds were excluded from the SSI classification [5]. 

The primary exposure variable was prophylactic antibiotic use. Additional baseline characteristics collected included sex, age, body mass index (BMI), hypertension status, diabetes mellitus, history of cancer, use of immunosuppressive agents, standard biochemical parameters (Albumin, aspartate transferase (AST), alanine transaminase (ALT), gamma-glutamyl transpeptidase (γ-GTP), hemoglobin (Hb), estimated glomerular filtration rate (eGFR)); these values were obtained from recent laboratory results when available, based on routine clinical practice at the time of consultation, as pre-biopsy blood testing was not uniformly conducted, the longest diameter of the wound, the type of biopsy specimen (benign or malignant), the biopsy site (head and neck, thorax, abdomen, back, genital, hip, upper limb, lower limb, sole), and the clinician's level of experience. In all cases, the physician who prescribed the antibiotic was the same as the one who performed the biopsy. A total of 17 physicians were included in the analysis.

Statistical analysis

Descriptive statistics were first generated to summarize both categorical and continuous variables. Fisher’s exact test was employed for categorical variables, while the Mann-Whitney U test was used for continuous variables. Univariate logistic regression was conducted to explore the association between individual covariates and SSI. Three multivariable logistic regression models were subsequently developed. Model 1 included prophylactic antibiotic use, age, BMI, albumin, and biopsy site as predictors. Model 2 was further adjusted for the clinician’s level of experience. Model 3 was constructed to examine factors associated with prophylactic antibiotic use, incorporating age, BMI, clinician experience, and albumin as explanatory variables. Localization was treated as a categorical variable in Models 1 and 2, but was not individually analyzed in the univariate analysis. As such, odds ratios (ORs) or 95% confidence intervals (CIs) were not calculated for each localization category; Instead, a p-value for the overall effect of localization on SSI risk was reported.

To account for potential confounding, inverse probability of treatment weighting (IPTW) was employed to balance covariates between the prophylactic antibiotic and non-antibiotic groups. Propensity scores were estimated through logistic regression, regressing prophylactic antibiotic use on age, BMI, albumin, and biopsy site. Standardized mean differences (SMD) were calculated before and after weighting to assess balance. IPTW-weighted logistic regression was then applied to evaluate the association between prophylactic antibiotic use and SSI, adjusting for potential confounders (age, BMI, albumin, biopsy site). A p-value of <0.05 was considered statistically significant. All statistical analyses were conducted using EZR (Easy R) version 1.53 (Saitama Medical Center, Jichi Medical University, Saitama, Japan) [13], a graphical user interface for R (The R Foundation for Statistical Computing, Vienna, Austria). 

Localization was included as a categorical variable in the logistic regression models, with the abdomen designated as the reference category. Due to limited sample sizes in certain localization categories, individual ORs and CIs should be interpreted with caution.

## Results

Patient characteristics

The baseline characteristics of the 202 patients included in this study are summarized in Table 1. Among them, 146 (72.3%) received prophylactic antibiotics, while 56 (27.7%) did not. Among the patients who received prophylactic antibiotics, 144 (98.6%) were prescribed cephalexin, while clarithromycin and levofloxacin were each prescribed to one patient (0.7%), respectively. The median age was 58.5 years (range, 11-95) for the antibiotic group and 67 years (range, 11-90) for the non-antibiotic group. The median BMI was 23.2 (range, 14.7-31.0) and 20.8 (range, 16.9-39.1), respectively. Histopathological examination of the biopsy specimens revealed that 44 patients (21.8%) had malignant tumors. Additionally, 35 (17.3%) had a prior history of cancer. Twenty patients (9.9%) had diabetes mellitus, and 14 (6.9%) were receiving immunosuppressive therapy, including prednisolone (n = 7), methotrexate (n = 4), tacrolimus (n = 2), and a Janus kinase inhibitor (n = 1). The overall incidence of SSI was 10.9% (22/202), with rates of 11.0% (16/146) in the prophylactic group and 10.7% (6/56) in the non-prophylactic group. Further characteristics, including laboratory findings, comorbidities, and biopsy site distributions, are detailed in Table 1.

**Table 1:** Baseline characteristics of patients in the prophylactic and non-prophylactic antibiotic groups Values are presented as median (range) for continuous variables and number (percentage) for categorical variables. AST: aspartate transferase; ALT: alanine transaminase; γ GTP: gamma-glutamyl transpeptidase; eGFR: estimated glomerular filtration rate; SSI: surgical site infection

Characteristics		Total Patients (n = 202)	Prophylactic Antibiotic group (n = 146)	Non-Prophylactic Antibiotic group (n = 56)	
					Sex, n (%)	Male	109 (54.0)	79 (54.1)	30 (53.6)	
Female	93 (46.0)	67 (45.9)	26 (46.4)		
Age (years)	median (range)	58.5 (11–95)	58 (11–95)	67 (11–90)	
BMI (kg/m^2^)	median (range)	22.5 (14.7–39.1)	23.2 (14.7–31.0)	20.8 (16.9–39.1)	
Type of tumor, n (%)	Benign	158 (78.2)	118 (80.8)	40 (71.4)	
	Malignant	44 (21.8)	28 (19.2)	16 (28.6)	
Duration of medication (days), median (range)			3 (1–21)	NA	
Blood test, median (range)	AST (U/L)	22 (10-100)	21 (13-55)	22.5 (10-100)	
	ALT (U/L)	18 (5-72)	16 (5-62)	23.5 (6-72)	
	γ GTP (U/L)	21.5 (8-361)	20 (8-350)	24 (9-361)	
	Hemoglobin (g/dL)	13.0 (5.5-17.0)	13.0 (8.7-17.0)	12.0 (5.5-16.9)	
	Albumin (g/dL)	4.1 (1.5-6.5)	4.1 (2.6-6.5)	3.8 (1.5-4.6)	
	eGFR (mL/min/1.73m²)	79.6 (25.1-139.9)	77.5 (25.1-127.1)	79.9 (28.7-139.9)	
Co-morbidities, n (%)	Hypertension	56 (27.7)	36 (24.7)	20 (35.7)	
	Diabetes mellitus	20 (9.9)	17 (11.6)	3 (5.4)	
	Cancer	35 (17.3)	22 (15.1)	13 (23.2)	
	Use of immunosuppressive agents	14 (6.9)	8 (5.5)	6 (10.7)	
Location of tumor, n (%)	Head and neck	32 (15.8)	31 (21.2)	1 (1.8)	
	Thorax	10 (5.0)	8 (5.5)	2 (3.6)	
	Abdomen	25 (12.4)	12 (8.2)	13 (23.2)	
	Back	43 (21.3)	37 (25.3)	6 (10.7)	
	Genital	3 (1.5)	3 (2.1)	0 (0)	
	Hip	15 (7.4)	11 (7.5)	4 (7.1)	
	Upper limb	25 (12.4)	16 (11.0)	9 (16.1)	
	Lower limb	44 (21.8)	23 (15.8)	21 (37.5)	
	Sole	5 (2.4)	5 (3.4)	0 (0)	
Physician's years of experience (years), median (range)		6 (2-18)	6 (2-18)	8.5 (2-14)	
Longest wound diameter (mm), median (range)		20 (6-55)	20 (7-55)	20 (6-35)	
SSI, n (%)		22 (10.9)	16 (11.0)	6 (10.7)	

Univariate analysis

Significant differences were observed in some continuous variables between the prophylactic and non-prophylactic groups. BMI was slightly higher in the prophylactic group than in the non-prophylactic group (23.2 vs. 20.8, p = 0.01). Serum albumin levels were also slightly higher in the prophylactic group (4.1 g/dL vs. 3.8 g/dL, p < 0.001). Conversely, AST levels were slightly higher in the non-prophylactic group (21 vs. 22.5, p = 0.04). Additionally, the median years of physician experience were lower in the prophylactic group (6.0 vs. 8.5, p = 0.002). Other variables did not demonstrate statistically significant differences (Table 2).

**Table 2:** Univariate analysis of continuous variables between the prophylactic and non-prophylactic antibiotic groups Continuous variables are presented as median (interquartile range). The Mann-Whitney U test was used to compare continuous variables between the prophylactic antibiotics group and the non-prophylactic antibiotic group. Median differences with 95% confidence intervals are also reported. * P < 0.05, ** P < 0.01, *** P < 0.001 AST: aspartate transferase; ALT: alanine transaminase; eGFR: estimated glomerular filtration rate; γGTP: gamma-glutamyl transpeptidase

Variable	Prophylactic Antibiotic group (n=146), median (IQR)	Non-Prophylactic Antibiotic group (n=56), median (IQR)	p-value	Median Difference (95% CI)
Age (years)	58 (46–74)	67 (50–75)	0.12	5.0 (-1.0–12.0)
BMI (kg/m^2^)	23.21 (20.38–24.37)	20.82 [18.99–22.93]	0.01*	-1.57 (-2.87–-0.43)
AST (U/L)	21 (18.00–25)	22.5 (18.25–43)	0.04*	4.00 (0.00–9.00)
ALT (U/L)	16 (12.0–24.5)	23.5 (13.25–28.0)	0.07	3.00 (0.00–8.00)
Hemoglobin (g/dL)	12 (10.6–14.0)	13 (12.4–14.1)	0.06	-0.9 (-1.8–0.0)
Albumin (g/dL)	4.1 (3.9–4.3)	3.8 (2.5–4.1)	< 0.001***	-0.40 (-0.80–-0.10)
γGTP (U/L)	20 (15–35)	24 (17–61)	0.13	4.00 (-1.00–10.00)
eGFR (mL/min/1.73m²)	77.5 (62.4–90.9)	79.9 (68.1–88.8)	0.58	1.96 (-6.32–11.33)
Physician's experience (years)	6 (4.00–8.00)	8.5 (5.75–12)	0.002**	2.00 (1.00–3.00)
Longest wound diameter (mm)	20 (15–25)	20 (15–22)	0.65	-0.00 (-2.00–1.00)

For categorical variables, no significant differences were noted between the two groups regarding sex, tumor type (malignant vs. benign), history of hypertension, cancer history, diabetes mellitus, or use of immunosuppressive agents. However, tumor localization differed significantly between the groups (p < 0.001) (Table 3). 

**Table 3:** Univariate analysis of categorical variables between the prophylactic and non-prophylactic antibiotic groups Categorical variables are presented as numbers (percentages). Fisher’s exact test was used to compare categorical variables between the prophylactic antibiotic group and the non-prophylactic antibiotic group. *NOTE: Localization was not analyzed individually in the univariate analysis. Therefore, no odds ratios (OR) or confidence intervals (CI) were calculated for each location category. SSI: surgical site infection *** P < 0.001, N/A: Not applicable

Variable	Category	Prophylactic Antibiotic group (n=146), n (%)	Non-Prophylactic Antibiotics group (n=56), n (%)	p-value	OR (95% CI)
Sex	Male	79 (54.1%)	30 (53.6%)	1	1.02 (0.52–1.98)
	Female	67 (45.9%)	26 (46.4%)		
Type of Tumor	Malignant	28 (19.2%)	16 (28.6%)	0.182	0.59 (0.28–1.30)
	Benign	118 (80.8%)	40 (71.4%)		
Hypertension	Yes	36 (24.7%)	20 (35.7%)	0.159	0.59 (0.29–1.22)
	No	110 (75.3%)	36 (64.3%)		
Diabetes mellitus	Yes	17 (11.6%)	3 (5.4%)	0.291	2.32 (0.63–12.87)
	No	129 (88.4%)	53 (94.6%)		
immunosuppressive agents	Yes	8 (5.5%)	6 (10.7%)	0.219	0.49 (0.14–1.78)
	No	138 (94.5%)	50 (89.3%)		
Location of tumor		See Note*	See Note*	< 0.001***	N/A
SSI	Yes	16 (11.0%)	6 (10.7%)	1	1.03 (0.36–3.39)
	No	130 (89.0%)	50 (89.3%)		

The SSI incidence was comparable between the two groups, at 11.0% in the prophylactic group and 10.7% in the non-prophylactic group, with no statistically significant difference (p = 1.0). As described in the Methods section, localization was included as a categorical variable in the multivariate analysis but was not individually analyzed in the univariate analysis. Therefore, no OR and 95% CI were calculated for each localization category. Instead, a p-value for the overall effect of localization on SSI risk was reported.

Multivariable logistic regression analysis (standard model)

Table 4 presents the results of the multivariable logistic regression analysis for SSI. The abdomen was selected as the reference category based on prior evidence suggesting that trunk skin surgeries pose a significantly lower infection risk compared to other anatomical sites (2.5%, n = 80/3,152 procedures, RR 0.75, 95%CI: 0.58-0.96) [14]. In addition, the abdomen had a relatively high case count and a low infection rate in our dataset, making it a suitable reference for comparison. The sole was excluded from the analysis due to the absence of SSI cases at this site. Variables included in the multivariable model were primarily selected based on significance in univariate analysis, along with established relevance to SSI risk, such as anatomical site and nutritional status. Other known risk factors for SSI, including diabetes mellitus and immunosuppressive therapy, were not incorporated due to the lack of statistical significance in univariate analysis and the limited number of affected patients, which may lead to model instability. None of the covariates, including prophylactic antibiotic use (OR 2.65, 95%CI: 0.38-18.44, p = 0.326), were significantly associated with SSI occurrence. Similarly, age, BMI, albumin levels, and biopsy site localization did not demonstrate any statistically significant influence on SSI risk.

**Table 4:** Multivariable logistic regression analysis (standard model) A multivariable logistic regression analysis was conducted to assess the association between prophylactic antibiotic use and the risk of surgical site infection. OR and 95%CI were calculated for each variable. Localization was analyzed using abdominal procedures as the reference category.

Variable	Odds ratio (95% CI)	p-value
Prophylactic Antibiotics	2.65 (0.38 – 18.44)	0.326
Age (years)	1.00 (0.95 – 1.05)	0.911
BMI (kg/m^2^)	0.84 (0.64 – 1.09)	0.191
Albumin (g/dL)	1.49 (0.30 – 7.47)	0.626
Location of the tumor		
Head and neck	1.47 (0.07 – 30.00)	0.803
Thorax	1.57 (0.05 – 48.07)	0.797
Genital	2.52 × 10⁸ (0 – ∞)	0.998
Back	1.28 (0.08 – 20.58)	0.86
Hip	0.76 (0.03 – 25.00)	0.869
Upper limb	7.55 × 10^−8 (0 – ∞)	0.993
Lower limb	0.63 (0.04 – 9.59)	0.74

IPTW analysis

To further address potential confounding, an IPTW-weighted multivariable logistic regression with robust standard errors was conducted (Table 5). Before IPTW adjustment, the SMD for age, albumin, BMI, and biopsy site were 0.238, 0.912, 0.337, and 1.082, respectively, indicating moderate imbalances. After IPTW adjustment, the SMDs improved to 0.044, 0.437, 0.153, and 0.661, respectively. Age achieved sufficient balance (SMD < 0.1), although slight imbalances persisted for albumin, BMI, and biopsy site.

**Table 5:** Multivariable logistic regression analysis after inverse probability of treatment weighting A multivariable logistic regression analysis was performed after applying inverse probability of treatment weighting (IPTW) to account for potential confounding variables. OR and 95%CI were calculated for each variable, with location of tumor analyzed using robust standard errors. The reference category for location was the abdomen. *** P < 0.001

Variable	Odds ratio (95% CI)	p-value
Prophylactic Antibiotic	3.95 (0.65 – 24.20)	0.137
Age (years)	0.99 (0.94 – 1.04)	0.63
BMI (kg/m^2^)	0.85 (0.69 – 1.04)	0.105
Albumin (g/dL)	1.29 (0.23 – 7.20)	0.77
Location of the tumor		
Head and neck	2.36 (0.17 – 32.00)	0.52
Thorax	1.80 (0.12 – 27.30)	0.671
Genital	4.52 × 10^8 (6.60 × 10^6 – 3.09 × 10^10)	2.39 × 10^−20***
Back	1.84 (0.18 – 18.50)	0.606
Hip	0.94 (0.03 – 27.10)	0.971
Upper limb	1.06 × 10^−7 (6.47 × 10^−9 – 1.74 × 10^−6)	2.15 × 10^−29***
Lower limb	0.81 (0.03 – 22.10)	0.898

Even after IPTW adjustment, prophylactic antibiotic use was not significantly associated with SSI (OR 3.95, 95%CI 0.65-24.20, p = 0.137). Similarly, none of the variables, including age, BMI, serum albumin, or biopsy site localization, demonstrated a significant association with SSI (p > 0.05 for all). The extreme ORs observed for the genital and upper limb localizations likely reflect the limited number of cases in these subgroups, resulting in quasi-complete separation, which should be interpreted cautiously.

Both the primary analysis and IPTW-adjusted findings consistently indicated no significant association between prophylactic antibiotic use and SSI incidence following elliptical skin biopsy. However, residual confounding cannot be entirely ruled out, given the slight remaining imbalances in some covariates.

Additional analysis, including physician experience

A multivariable logistic regression model was developed, incorporating physician years of experience as an additional covariate, as it was initially significant in the univariate analysis (Model 2). However, in this expanded model, physician experience was not significantly associated with SSI risk (OR 1.09, 95%CI 0.83-1.43, p = 0.57), and the overall results remained consistent with our primary analysis (Table 6). Given the limited sample size and the established role of other key factors, such as biopsy site, BMI, and serum albumin, in infection risk, we decided to retain only these core variables in the final model.

**Table 6:** Multivariable logistic regression analysis (standard model including years of experience) A multivariable logistic regression analysis was conducted to assess the association between prophylactic antibiotic use and the risk of surgical site infection (SSI). Odds ratios (OR) and 95% confidence intervals (CI) were calculated for each variable. Localization was analyzed using abdominal procedures as the reference category.

Variable	Odds ratio (95% CI)	p-value
Prophylactic Antibiotic	3.48 (0.38 – 31.8)	0.27
Age (years)	1.00 (0.95 – 1.05)	0.91
BMI (kg/m^2^)	0.81 (0.61 – 1.09)	0.16
Albumin (g/dL)	1.62 (0.29 – 9.14)	0.58
Physician's experience (years)	1.09 (0.81 – 1.47)	0.57
Localization		
Head and neck	1.53 (0.074 – 31.6)	0.78
Thorax	1.12 (0.031 – 41.0)	0.95
Genital	2.23 × 10^8 (0 – ∞)	1.00
Back	1.22 (0.074 – 20.1)	0.89
Hip	0.61 (0.021 – 17.7)	0.77
Upper limb	8.14 × 10^−8 (0 – ∞)	0.99
Lower limb	0.69 (0.044 – 10.7)	0.79

Factors associated with prophylactic antibiotic use

To further explore factors influencing the prescription of prophylactic antibiotics, a separate multivariable logistic regression model (Model 3) was conducted, considering age, BMI, albumin levels, and clinician years of experience as predictors. The analysis revealed that higher albumin levels were significantly associated with greater use of prophylactic antibiotics (OR 5.23, 95%CI 1.84-14.90, p = 0.002), while increased clinician experience was significantly associated with a lower use (OR 0.75, 95%CI 0.61-0.91, p = 0.0042) (Table 7).

**Table 7:** Multivariable logistic regression analysis (association with prophylactic antibiotics use) A multivariable logistic regression analysis was conducted to assess the association between prophylactic antibiotic use and patient characteristics. OR and 95%CI were calculated for each variable. ** P < 0.01

Variable	Odds ratio (95% CI)	p-value
Age (year)	1.01 (0.98 – 1.05)	0.45
Albumin (g/dL)	5.23 (1.84 – 14.9)	0.002**
BMI (kg/m^2^)	1.14 (0.96 – 1.34)	0.13
Years of experience	0.75 (0.61 – 0.91)	0.004**

## Discussion

A retrospective study was conducted to evaluate whether prophylactic antibiotic administration contributed to reducing the incidence of SSI following elliptical biopsy in a single-institution setting. Among patients who underwent elliptical biopsy at our institution, 72.3% (146 out of 202 patients) received postoperative prophylactic antibiotics, a proportion higher than previously reported rates [9], likely reflecting institutional practices or variations in patient characteristics. Given that university hospitals often manage complex cases, including malignancies, and provide multidisciplinary care, prophylactic antibiotic use may have been more commonly adopted in our setting.

The overall incidence of SSI was 10.9% (22 out of 202 patients), consistent with rates reported in similar settings [15,16]. Despite the relatively high proportion of patients receiving prophylactic antibiotics, the actual incidence of SSI did not show a substantial reduction. To further examine factors influencing prophylactic antibiotic use, we performed a multivariable logistic regression analysis incorporating clinician experience as an explanatory variable (Model 3). 

The results indicated that higher serum albumin levels were significantly associated with greater use of prophylactic antibiotics, while greater clinician experience was significantly associated with lower use. In other words, clinicians with fewer years of experience were more likely to prescribe prophylactic antibiotics. Serum albumin is recognized as an inflammatory marker associated with nutritional risk [17,18]. In this study, patients with higher serum albumin levels were more likely to receive prophylactic antibiotics. This finding suggests that antibiotics may have been prescribed even in the absence of significant nutritional or inflammatory risk, indicating that prescribing decisions may not have been based on objective clinical indicators such as inflammatory status. Although the difference in albumin levels between the groups (4.1 vs. 3.8 g/dL) was statistically significant, the magnitude of the difference was small and unlikely to be clinically meaningful. Therefore, this association should be interpreted with caution. The findings are more likely to reflect subjective factors influencing prescribing behavior, such as physician experience, institutional norms, or generalized concern about SSI, rather than a true biological relationship. In particular, less experienced physicians may have prescribed antibiotics as a form of psychological reassurance, whereas more experienced clinicians appeared to make more selective and judgment-based decisions.

Routine postoperative antibiotic administration may not be essential for SSI prevention following elliptical biopsy. Although this study was retrospective and subject to inherent selection biases, we adjusted for potential confounders using logistic regression analysis and IPTW. Notably, even after IPTW adjustment, the p-value remained non-significant, indicating that the absence of a clear association between prophylactic antibiotic use and SSI was not merely due to baseline imbalances. Under typical conditions of elliptical biopsy, prophylactic antibiotic use does not appear to have a decisive impact on reducing SSI risk.

Our findings highlight the importance of exploring alternative strategies to minimize infection risk while ensuring safe wound healing. Emphasizing standard infection prevention measures, such as meticulous wound irrigation, proper postoperative care, and patient education on wound management, may be more effective and sustainable than routine prophylactic antibiotic use.

While studies on the comparative efficacy of detergents and antiseptics, including povidone-iodine, in postoperative wound care are limited, existing evidence suggests that wound irrigation with running water or soap cleansing is sufficient in the absence of infection signs [19,20]. 

Routine use of topical antibiotics for clean excision wounds is generally not recommended, as current evidence does not support a reduction in SSI incidence, and there is a potential risk of adverse effects such as contact dermatitis [21-23]. Although evidence is lacking, topical antibiotics cannot be entirely ruled out in exceptional circumstances. Regarding early postoperative bathing, including showering, meta-analyses have indicated that it has been shown to enhance patient comfort and is considered a safe practice [24]. Covering the wound for the initial 48 hours is recommended [19]. Foam dressings, which require infrequent changes, are often preferred, although they may lead to complications such as exudate pooling, backflow, or leakage, increasing the risk of skin damage, infections, and other wound-related issues [25]. Therefore, careful selection and appropriate use of dressings are critical. Providing patients with concise yet comprehensive education on hand hygiene, wound care, and appropriate antibiotic use can foster adherence to infection prevention measures, ultimately improving postoperative outcomes [26].

Japan faces challenges concerning antibiotic availability and pricing regulations. The government-regulated pricing system has contributed to recurrent shortages of essential antibiotics such as cefazolin sodium [27], primarily due to prolonged price reductions and insufficient financial incentives for pharmaceutical companies to maintain production [28,29].

This issue is not confined to Japan; antibiotic shortages have also been reported in Western countries due to manufacturing constraints, supply chain disruptions, and low profitability [28,29]. These circumstances underscore the necessity of optimizing antibiotic use, particularly in minor dermatologic surgeries. The financial sustainability of antibiotic production has been widely discussed as a global concern, with declining investment in antimicrobial development due to limited profitability [30]. Reevaluating the need for prophylactic antibiotics in procedures such as elliptical biopsy is vital for antimicrobial stewardship and addressing supply challenges.

Limitations of the study

This study has several limitations. As a retrospective observational study, it is subject to potential biases such as selection bias and unmeasured confounding. Although we employed multivariable logistic regression and IPTW to adjust for known confounders, residual confounding cannot be entirely excluded. The decision to prescribe prophylactic antibiotics was made at the discretion of individual clinicians without a standardized institutional protocol, potentially introducing variability in prescribing practices. Clinician experience and patient characteristics, including serum albumin levels, were statistically associated with antibiotic prescription decisions; however, these associations should be interpreted with caution, as they may reflect background tendencies rather than causal relationships. In addition, the relatively small sample size (n = 202) may have limited the statistical power to detect modest but clinically relevant associations. Subgroup analyses of certain biopsy sites, such as the genital or upper limb, yielded extreme odds ratios and wide confidence intervals, suggesting statistical instability. Furthermore, because the definition of SSI was limited to a 30-day postoperative period, long-term outcomes could not be assessed. Nevertheless, our findings consistently suggest that prophylactic antibiotic use was not significantly associated with a reduction in SSI risk, supporting the need to reconsider routine antibiotic use following elliptical skin biopsy. Further prospective studies with larger sample sizes and standardized treatment protocols are warranted to better define the role of prophylactic antibiotics in this setting.

## Conclusions

This retrospective study did not identify a significant association between prophylactic antibiotic use and reduced SSI risk following elliptical skin biopsy. Although these findings do not conclusively determine the necessity or futility of prophylactic antibiotics, they underscore the importance of reevaluating their routine use in dermatologic procedures.

Given global concerns about antimicrobial resistance and antibiotic supply constraints, optimizing antibiotic use is imperative. While further prospective studies are needed to validate these findings, the current study suggests that prophylactic antibiotics may not be universally required for elliptical biopsy. Dermatologists should carefully balance the potential benefits of antibiotic prophylaxis against its implications for antimicrobial stewardship and patient safety.
